# SARS-CoV-2 Evolution: On the Sudden Appearance of the Omicron Variant

**DOI:** 10.1128/jvi.00090-22

**Published:** 2022-03-16

**Authors:** Ben Berkhout, Elena Herrera-Carrillo

**Affiliations:** a Department of Medical Microbiology, Amsterdam University Medical Centers, University of Amsterdam, Amsterdam, the Netherlands; Loyola University—Chicago

**Keywords:** evolution, Omicron, SARS-CoV-2

## Abstract

The Omicron variant of severe acute respiratory syndrome coronavirus 2 (SARS-CoV-2) spreads rapidly and harbors many mutations in the spike protein, but the origin of this virus variant remains unclear. We address the role of unusual virus evolution mechanisms such as hypermutation, out-of-frame reading, and recombination. Rather, regular Darwinian evolution, that is, the repeated selection of beneficial spike mutations, seems to have led to the appearance of the grossly altered spike protein of the Omicron variant.

## TEXT

Severe acute respiratory syndrome coronavirus 2 (SARS-CoV-2) is the virus that causes coronavirus disease 2019 (COVID-19). This virus unleashed a true pandemic in 2019 that continues until today, although a fair level of protection can be achieved by several newly developed vaccines and, hopefully, by herd immunity. Multiple SARS-CoV-2 variants of concern (VOCs) have been designated by the World Health Organization (WHO). Omicron (B.1.1.529) is the newest virus variant first reported from South Africa in November 2021 (1, 2). Compared to other VOCs, Omicron is believed to spread much quicker, as first shown for South Africa and recently confirmed for the United Kingdom and other countries (3–6). The Omicron variant has acquired around 60 mutations compared to the original Wuhan isolate, a finding that has concerned scientists. Some 35 mutations can be found in the gene encoding the spike protein that result in 30 amino acid substitutions (Table 1) (7). This was recently described as an unusual cluster of mutational changes, suggesting that initial mutations may have caused a fitness loss, which triggered additional but compensatory changes to mitigate the fitness loss (8). The spike protein is responsible for attachment to target cells, and the receptor-binding domain (RBD) runs from positions 319 to 541. The spike protein also facilitates subsequent viral entry into the cell, and the many Omicron substitutions may thus influence the cell tropism and pathogenicity of SARS-CoV-2. In fact, any change in the amino acid side chain (charge, size, and hydrophobicity) can have an effect on the intrinsic properties of the affected protein and on the interaction with other proteins or other molecules. The Omicron strain can replicate rapidly *in vitro* on human primary nasal epithelial cultures (9). The spike protein is the only viral protein that decorates the surface of virion particles and is the main antigenic target of antibodies induced by natural infection and vaccination. Thus, another concern is that the Omicron spike protein may be less sensitive to the current vaccines, although rapid adaptation of especially the RNA-based vaccines seems fairly easy.

**TABLE 1:** Mutations acquired by SARS-CoV-2 Omicron compared to the Wuhan prototype^*a*^

Mutation	Codon change	Substitution	Peculiarity
21762 C to U	GCU to GUU	Ala67Val	
21765–21770 ΔUACAUG			6 nt/2 aa removed
21846 C to U	ACU to AUU	Thr95Ile	
21987–21995 ΔGUGUUUAUU			9 nt/3 aa removed
22194–22196 ΔAUU			3 nt/1 aa removed
22205 +GAGCCAGAA			9 nt/3 aa inserted
22587 G to A	GGU to GAU	Gly339Asp	Charge gain −1
22599 G to A	AGA to AAA	Arg346Lys	
22673 UC to CU	UCC to CUC	Ser371Leu*	
22679 U to C	UCA to CCA	Ser373Pro*	
22686 C to U	UCC to UUC	Ser375Phe*	
22813 G to U	AAG to AAU	Lys417Asn	Charge loss +1
22882 U to G	AAU to AAG	Asn440Lys	Charge gain +1
22898 G to A	GGU to AGU	Gly446Ser	
22992 G to A	AGC to AAC	Ser477Asn*	
22995 C to A	ACA to AAA	Thr478Lys*	Charge gain +1
23013 A to C	GAA to GCA	Glu484Ala	Charge loss −1
23040 A to G	CAA to CGA	Gln493Arg*	Charge gain +1
23048 G to A	GGU to AGU	Gly496Ser*	
23055 A to G	CAA to CGA	Gln498Arg*	Charge gain +1
23063 A to U	AAU to UAU	Asn501Tyr*	
23075 U to C	UAC to CAC	Tyr505His*	Charge gain +1
23202 C to A	ACA to AAA	Thr547Lys	Charge gain +1
23403 A to G	GAU to GGU	Asp614Gly	Charge loss −1
23525 C to U	CAU to UAU	His655Tyr	Charge loss +1
23599 U to G	AAU to AAG	Asn679Lys	Charge gain +1
23604 C to A	CCU to CAU	Pro681His	Charge gain +1
23664 C to U	GCA to GUA	Ala701Val	
23854 C to A	CCG to ACG	Pro764Thr	
23948 G to U	GAU to UAU	Asp796Tyr	Charge loss −1
24130 C to A	AAC to AAA	Asn856Lys	Charge gain +1
24424 A to U	CAA to CAU	Gln954His	Charge gain +1
24469 U to A	AAU to AAA	Asn969Lys	Charge gain +1
24503 C to U	CUU to UUU	Leu981Phe	
25000 C to U	GAC to GAU	Silent	

a nt, nucleotides; aa, amino acids; +, insertion; Δ, deletion; *, mutation cluster (<10 bp between two mutations).

The origin of the Omicron variant remains unclear (10, 11). A curious feature of Omicron is that it consists of three distinct sublineages (called BA.1, BA.2, and BA.3) that seem to have emerged at around the same time (8, 12). Two of these lineages have taken off globally. Anyhow, what this means is that Omicron had time to diversify before it was discovered. In this brief perspective, we focus on the potential mechanisms of virus evolution that led to the Omicron variant. The standard scenario of virus evolution calls for the repeated selection of spontaneous mutations that affect the phenotype of the virus, e.g., by improved recognition of the receptor, a switch to other host cells, or being less visible to the innate and adaptive immune systems. This scenario of Darwinian evolution seems quite possible for spike adaptations in SARS-CoV-2 as this protein not only mediates the recognition of the ACE2 receptor on target cells to trigger virus entry but also is the prime target for antibodies. This means that there is much selection pressure acting on the viral spike protein, which could explain the acquisition of many mutations in the spike gene of the Omicron isolate.

The acquisition of many clustered mutations in the spike gene could in theory also be explained by more exotic evolutionary events that we will introduce and critically test. First, viral genomes can be subject to “hypermutation,” a process in which multiple changes occur in a clustered manner in parts of the viral genome. These mutations are introduced either by the viral polymerase, possibly by an error-prone mutant thereof, or by cellular restriction systems as exemplified by the HIV apolipoprotein B mRNA-editing catalytic polypeptide-like protein (APOBEC) case (13, 14). A characteristic of hypermutation is that a common mutational pattern can usually be recognized, e.g., monotonous G-to-A changes in the HIV genome (15). Mutation pressure may also have been exerted on the SARS-CoV-2 RNA genome by antiviral defense systems of the host cell, including APOBEC, adenosine deaminase acting on RNA proteins (ADAR), and reactive oxygen species (ROS) (16). The resulting hypermutation signatures will not always remain visible as they may become blurred during the evolutionary path from the parental virus strain to Omicron. We inspected the required mutations assuming a direct evolutionary path from the prototype Wuhan strain to the new Omicron variant (Table 1). The scenario may be too simple, but a more precise picture should await the identification of intermediate variants in the evolutionary history of Omicron. We analyzed the mutations that are common among 629 Omicron isolates. The sequences were extracted from the GISAID EpiCoV database on 8 December 2021. We excluded nucleotide changes that were described for fewer than 10 isolates. Therefore, the listed mutations do not represent random isolate-specific mutations but represent truly acquired characteristics of the Omicron lineage. No distinct mutational profile was observed (Table 1). Instead, we scored a regular pattern with more transitions (6 C-to-U, 5 G-to-A, 2 U-to-C, and 3 A-to-G changes) than transversions (5 C-to-A, 2 G-to-U, 2 U-to-G, 2 A-to-U, 1 A-to-C, 1 U-to-A, and no C-to-G and G-to-C changes), in total 16 transitions versus 13 transversions. The absence of any particular mutational profile does not support the hypermutation scenario. However, even a mild mutational bias, e.g., the prevalence of mutations that remove C residues, can, when executed over an extended evolutionary time scale, contribute to the biased nucleotide composition that can be recognized in all coronavirus RNA genomes (17).

A second evolutionary scenario that could instantaneously generate many mutations is a double-frameshift event that causes the ribosome to temporarily read in a different reading frame (18). This out-of-frame reading should be restricted to a short segment of the spike open reading frame as extended out-of-frame reading will likely be lethal because inactive proteins will be made or premature stop codons will soon be encountered. Inspection of the Wuhan-to-Omicron changes revealed a single insertion of 9 nucleotides that adds 3 amino acids, and three small deletions were present in the N-terminal domain of the spike protein that caused the removal of 1, 2, and 3 amino acids. Theoretically, a certain combination of deletion plus insertion could cause temporary out-of-frame reading; but all four indel mutations in Omicron are in frame, and this second scenario can thus also be refuted. The remarkable clustering of an insertion and multiple deletions may eventually tell us something about the structure or function of this small domain of the spike protein.

To broaden the test of the first two hypotheses, we also inspected the hypothetical evolutionary paths from any of the VOCs (Alpha, Beta, Delta, Delta plus, and Gamma) to Omicron. This analysis also did not reveal any special mutational profile or frameshift events that could support these scenarios (results not shown).

A third scenario that can trigger a gross yet sudden mutational event is recombination, the mixing of Omicron genome sequences with sequences derived from other viruses or the human host. Such swapping of genetic information is a well-known phenomenon in virology and is well documented for coronaviruses (19). For instance, it was recently proposed that Omicron acquired a 9-nucleotide stretch from the common cold coronavirus 229E (20). In general, there is currently no good evidence to support this evolutionary scenario, but the future identification of evolutionary intermediates of the Wuhan-to-Omicron transition may provide more definitive answers.

Having considered three unusual scenarios for the evolution of the Omicron variant, we cautiously conclude that regular virus evolution, that is, by the repeated selection of beneficial mutations in the spike gene or Darwinian evolution, underlies the appearance of this variant. Because Omicron first appeared in South Africa, it has been suggested that SARS-CoV-2 could evolve rapidly in this setting because of the weakened immune system of more than 20% of the local human population that is HIV infected (21). In light of recent reports of rapid SARS-CoV-2 evolution in unsuppressed HIV patients, most of the current discussion centers around chronic infections in immunocompromised hosts (22). But other hypothetical contributors include a variety of causes: selective pressure exerted by vaccination; the impact of modification of antigenic sites in spike on receptor recognition and viral fitness; the very likely important, yet ill-understood, determinants of virus transmissibility; and the impact of genetic bottlenecks that may occur during virus transmission.

The most compelling evidence for this scenario of regular Darwinian evolution actually comes from inspection of the genetic changes, which reveals a profound preference for mutations that change the amino acid composition of the spike protein: 30 nonsilent changes versus 1 silent mutation (Table 1). This evolutionary pressure is also reflected in the type of codon change observed (Table 1): 14 at the second codon position, 9 at the first codon position, and only 7 with a change in the third codon position that frequently creates a synonymous codon that does not change the encoded amino acid. In fact, 6 of the latter 7 codon changes are nonsilent, underlining the idea that strong selection pressure acted on the Omicron spike protein.

Some or all of these amino acid substitutions may affect the basic viral functions of receptor recognition and cell entry, and other substitutions may represent compensatory changes in the spike protein. We noticed a remarkable adjustment in the usage of charged amino acids: 2 positively charged residues were removed, but some 11 were gained, whereas a more balanced picture was seen for negatively charged residues (1 gained and 3 removed). Such changes in net or local charge could affect the interactions of the spike protein with other molecules, among which are the ACE2 receptor and antibodies raised by natural infection or immunization. Experimental studies are needed to test these ideas. Mutations in key domains of the spike protein may have an impact on the virulence and pathogenicity of this virus, possibly correlating with early epidemiological and clinical data showing that Omicron is less pathogenic than other SARS-CoV-2 variants (23).

In conclusion, it seems likely that strong evolutionary pressure has led to the selection of the grossly altered spike protein of the Omicron variant. However, much remains currently unknown. First of all, extensive sampling may reveal virus isolates that are intermediate between the Wuhan and Omicron strains, which could more firmly prove or deny the exact path of virus evolution. Experiments should then address the biological effects imposed by the many spike protein substitutions, both direct effects on virus replication and also indirect effects through escape from host-induced immune pressure. In the meantime, we should rapidly update the RNA-based vaccines against this rapidly spreading SARS-CoV-2 variant.
